# Prion subcellular fractionation reveals infectivity spectrum, with a high titre-low PrP^res ^level disparity

**DOI:** 10.1186/1750-1326-7-18

**Published:** 2012-04-26

**Authors:** Victoria Lewis, Cathryn L Haigh, Colin L Masters, Andrew F Hill, Victoria A Lawson, Steven J Collins

**Affiliations:** 1 Department of Pathology, The University of Melbourne, Parkville, VIC 3010, Australia; 2 Mental Health Research Institute and the Melbourne Brain Centre, The University of Melbourne, Parkville, VIC 3010, Australia; 3 Department of Biochemistry and Molecular Biology and the Bio21 Molecular Science and Biotechnology Institute, The University of Melbourne, Parkville, VIC 3010, Australia

**Keywords:** Prion protein, Prion infectivity, Prion disease, Protease resistance, Subcellular localisation, Fractionation

## Abstract

**Background:**

Prion disease transmission and pathogenesis are linked to misfolded, typically protease resistant (PrP^res^) conformers of the normal cellular prion protein (PrP^C^), with the former posited to be the principal constituent of the infectious 'prion'. Unexplained discrepancies observed between detectable PrP^res ^and infectivity levels exemplify the complexity in deciphering the exact biophysical nature of prions and those host cell factors, if any, which contribute to transmission efficiency. In order to improve our understanding of these important issues, this study utilized a bioassay validated cell culture model of prion infection to investigate discordance between PrP^res ^levels and infectivity titres at a subcellular resolution.

**Findings:**

Subcellular fractions enriched in lipid rafts or endoplasmic reticulum/mitochondrial marker proteins were equally highly efficient at prion transmission, despite lipid raft fractions containing up to eight times the levels of detectable PrP^res^. Brain homogenate infectivity was not differentially enhanced by subcellular fraction-specific co-factors, and proteinase K pre-treatment of selected fractions modestly, but equally reduced infectivity. Only lipid raft associated infectivity was enhanced by sonication.

**Conclusions:**

This study authenticates a subcellular disparity in PrP^res ^and infectivity levels, and eliminates simultaneous divergence of prion strains as the explanation for this phenomenon. On balance, the results align best with the concept that transmission efficiency is influenced more by intrinsic characteristics of the infectious prion, rather than cellular microenvironment conditions or absolute PrP^res ^levels.

## Background

Prion diseases constitute a group of unique neurodegenerative disorders, which naturally afflict a number of mammalian species including humans. Although our understanding remains incomplete, considerable evidence supports the "protein-only" hypothesis, which purports that the agent ("prion") responsible for both transmission and consequent pathogenesis is predominantly composed of misfolded conformers of the normal cellular prion protein PrP^C ^[1]. Additional discriminating features of the aberrant prion protein include increased β-sheet content [2,3], reduced solubility and increased tendency to aggregate, and typically heightened protease resistance [4-6]. Due to the characteristic protease resistant core, limited proteolysis with proteinase K (PK) truncates the N-terminus of the misfolded protein, producing PrP^res^, whilst PrP^C ^is completely degraded, allowing a convenient biochemical differentiation of these two prion protein isoforms.

An intriguing but somewhat perplexing aspect of prion biology is the several instances in transmission studies, encompassing many prion strains, where infectivity titres and PrP^res ^levels (as detected by biochemical assessment of inocula) do not faithfully correlate. Illustrating this are pre-clinical prion infections after low dose transmissions [7], BSE infectivity in tongue and nasal mucosa [8] and slowly sedimenting high titres of infectivity separated from PrP^res ^in 'fast' prion strains [9]. Further examples have occurred during cross species transmissions including intracerebral inoculation of hamster prions to mice [10], primary passage of bovine prions to rodents [11,12], scrapie prions peripherally introduced into mice [13] and transmission of three distinct prion strains (human, hamster scrapie, murine scrapie) into transgenic mice expressing the murine equivalent of a human prion protein gene mutation [14]. In addition, PrP^res ^generated through protein misfolding cyclic amplification (PMCA) evinces a longer incubation period (indicative of a lower titre) despite western blot detection levels equivalent to those observed in the original seeding inoculum [15]. This PMCA study suggests that a component within the original inoculum, which perhaps does not propagate or amplify as well as PrP^res^, may contribute to the more efficient transmission. Although PrP^res ^is inextricably linked to prion infectivity, these numerous examples clearly illustrate the poorly understood complexities of this relationship.

The precise cellular location of PrP^C ^misfolding and conversion also remains speculative (reviewed in [16]), as does the contribution of cellular co-factors to conversion efficiency, although the participation of a species-specific protein [17-19], or negatively charged macromolecules such as nucleic acids [20-24] and glycosaminoglycans [25-29] has been posited. In contrast, evidence exists correlating the efficiency of prion propagation and transmission with the size of prion multimers serving as templates for conversion [30,31]. Acknowledging the aforementioned uncertainties, the current study investigated whether such observed disparities between infectivity titres and PrP^res ^levels could be resolved to a subcellular level and thereby provide a useful model for insights into the molecular basis of this observation. To address this aim, we utilized fractionation of MoRK13 cells infected with M1000 prions to explore the contributions of subcellular co-factors and cognate prion protein species to the efficiency of transmission.

## Results

### Prion protein conformers reside predominantly in lipid rafts in MoRK13 and MoRK13-inf cells

**Figure 1:**
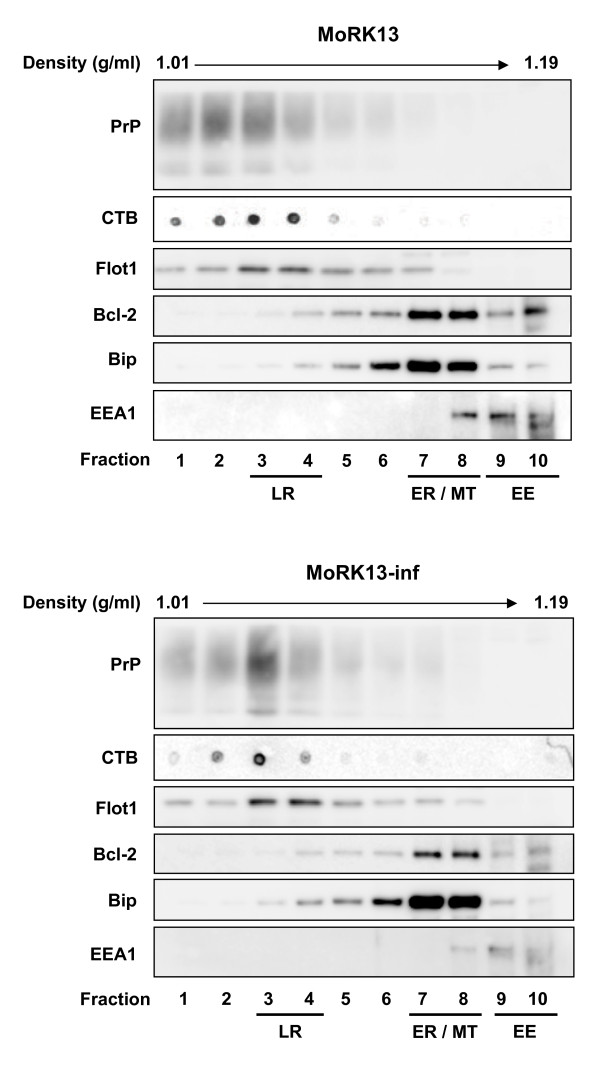
**MoRK13 and MoRK13-inf PrP localises predominantly to lipid raft fractions**. Representative immunoblots of subcellular fractions (1-10) obtained from MoRK13 and MoRK13-inf. Equivalent volumes of each fraction were resolved on gradient gels. Proteins were transferred to PVDF, which was sectioned based on the predicted molecular weight of the protein of interest, with each PVDF strip probed with the relevant antibody. CTB dot blot - 3 μl of fraction was dried onto nitrocellulose membrane, and blotted as described in the methods. LR = lipid raft; ER = endoplasmic reticulum; MT = mitochondria; EE = early endosome; CTB = cholera toxin subunit B (LR marker); Flot1 = Flotillin 1 (LR marker); Bcl-2 = anti-apoptotic MT marker protein; Bip = ER lumen chaperone protein; EEA1 = EE antigen 1.

**Figure 2:**
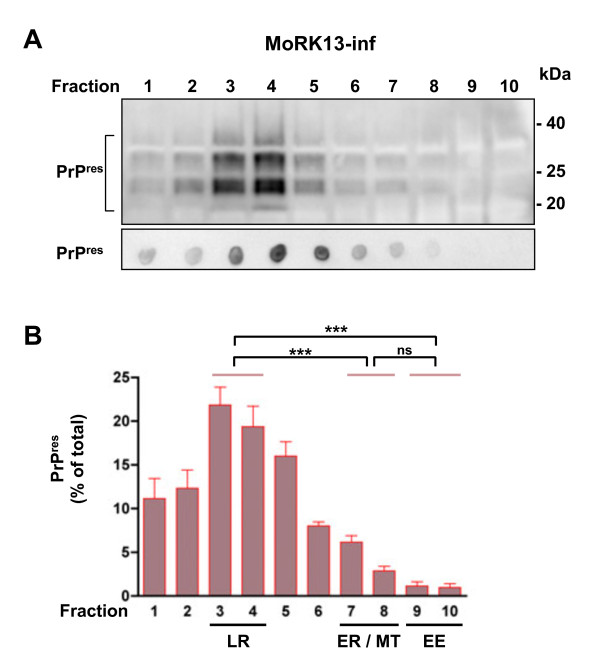
**PrP^res ^is enriched in lipid raft fractions of MoRK13-inf cells**. (A) Equivalent volumes of PK treated MoRK13-inf fractions resolved on 10-20% Tris-glycine SDS PAGE and immunoblotted (top panel), and representative fraction PrP^res ^dot blot (bottom panel). (B) Quantification of PrP^res ^from dot blots of six separate cellular fractionations. Fractions enriched in lipid raft (LR), endoplasmic reticulum (ER), mitochondrial (MT) and early endosome (EE) marker proteins are highlighted. Comparison of relative PrP^res ^levels by one way ANOVA; *** p < 0.001, ns = not significant.

Characterisation of the MoRK13-M1000 prion infection model (MoRK13-inf) determined that per detectable 'PrP^res ^unit', MoRK13-inf cell lysate was approximately 90 times more efficient than crude M1000 brain homogenate at prion transmission to recipient MoRK13 (Additional file 1: Figure S1). This finding, perhaps an example of strain adaptation into a rabbit cell line, highlighted a discrepancy between PrP^res ^and infectivity levels, and indicated that MoRK13-inf cells may be an appropriate model to study this phenomenon. PrP isoforms have been shown to localise to various subcellular environments, including those of the secretory and endocytic pathways, and at the cell surface [32-34]. Figure 1 shows the localisation of organelle markers Bcl-2 (mitochondria; MT), Bip (endoplasmic reticulum; ER), and EEA1 (early endosome; EE) in the MoRK13 cells. As expected, the lipid raft marker Flotillin 1 was enriched within fractions at the buoyant end of the gradient, consistent with the high cholesterol:protein ratio resulting in a significantly lower density of lipid rafts compared to other solubilised membrane proteins [35]. This was confirmed by dot blot analysis of the lipid raft marker GM_1_, detected by the cholera toxin subunit B (CTB). Figure 1 also indicates that the subcellular localisation of PrP^C ^in MoRK13, and total PrP (PrP^C ^and PrP^res^, indistinguishable from each other under the conditions of these western blots) in MoRK13-inf is predominantly in the buoyant, lipid raft fractions. There were no substantial differences in organelle or membrane markers or PrP localisation when comparing the MoRK13 and MoRK13-inf cells, and also comparing vector-only transfected RK13 (vecRK13), GT1-7H and GT1-7H-inf cells (Additional file 2: Figure S2); therefore over-expression of PrP^C^, prion infection and cell line origin (neuronal/non-neuronal/murine/rabbit) do not appear to alter localisations, or create artefacts in this system of subcellular fractionation. Previous reports indicate PrP^res ^is also localised in lipid rafts [36]. Figure 2A shows the localisation of MoRK13-inf PrP^res ^to be principally in lipid raft fractions, with a similar distribution to total PrP (Figure 1) as determined by both western blot and dot blot. Quantification of relative PrP^res ^levels (Figure 2B) highlights that the lipid raft enriched fractions contain up to eight times the detectable PrP^res ^levels than the ER/MT marker enriched fractions (eg compare fractions #3 and #8). By way of comparison to the original prion strain, Nycodenz gradient distribution of PrP isoforms and marker proteins from M1000 brain, homogenised in a comparative manner, was also similar to that observed in cell lysates (Additional file 3: Figure S3).

### High levels of prion infectivity are present in lipid raft and ER/MT marker enriched fractions of MoRK13-inf cells

**Figure 3:**
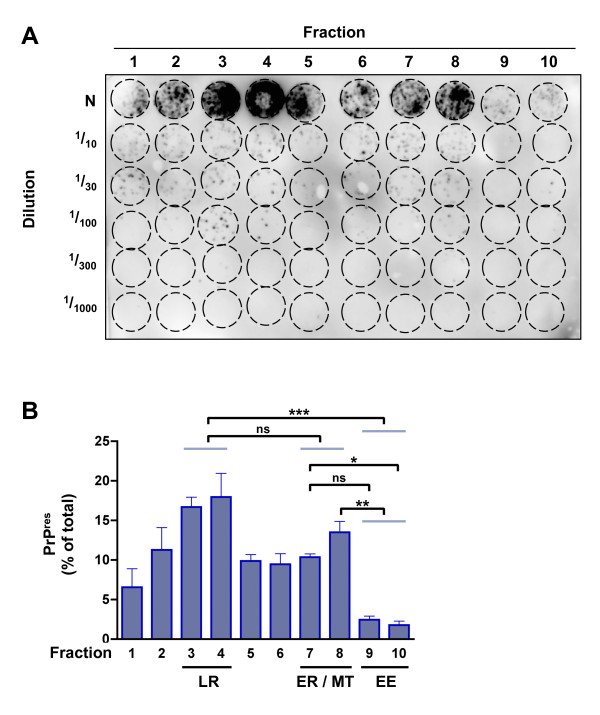
**All MoRK13-inf fractions contain infectivity**. (A) Representative cell blot showing PrP^res ^levels produced by recipient MoRK13 cells at passage 4 (P4) post-exposure to equal volumes of each fraction at the 'neat' dilution (N) or further serially diluted (1/10-1/1000). (B) Quantification of infectivity from three independent MoRK13-inf fractionations (n = 3); fractions enriched in lipid raft (LR), endoplasmic reticulum (ER), mitochondrial (MT) and early endosome (EE) marker proteins are highlighted. Statistical analysis by one way ANOVA; only the statistical findings for relevant comparisons are indicated: *** p < 0.001, ** p < 0.001, * p < 0.05, ns = not significant.

In order to determine whether detectable subcellular PrP^res ^levels correlate with infectivity, an *in vitro *cell culture transmission study was carried out of equivalent volumes of fractions obtained after density gradient flotation assays of MoRK13-inf cell lysate using the highly susceptible MoRK13 as recipient cells. Levels of PrP^res ^produced by recipient cells after exposure to fractions were quantified and used as a surrogate indicator of relative infectivity titres contained within the subcellular fractions. The representative cell blot and quantification (Figure 3A, B) clearly show that infectivity was contained within all fractions derived from MoRK13-inf cells, albeit to varying degrees. The EE enriched fractions (predominantly #9 and #10) contained considerably less infectivity compared with the lipid raft enriched fractions (predominantly #3 and #4), and the ER and MT marker enriched fractions (predominantly #7 and #8), although the comparison of fraction #7 and fraction #9 did not reach significance. Interestingly, there was no significant difference in the amount of infectivity contained within the lipid raft enriched fractions (#3 and #4), and the ER and MT marker enriched fractions (#7 and #8). As shown in Figure 3A, fractions were also diluted in a 1/2 log series, to ensure there was no limiting sensitivity of the *in vitro *assay truncating the upper end of the dynamic range. Importantly, neat fractions that elicited the highest production of PrP^res ^by recipient MoRK13 cells (for example fractions #4 and #8), were infectious to the same dilutions (1:100), thereby validating the use of PrP^res ^produced by recipient cells as a measure of relative infectivity contained within fractions.

### ER and MT marker enriched fractions show disparity between PrP^res ^levels and infectivity titres *in vitro *and *in vivo*

**Figure 4:**
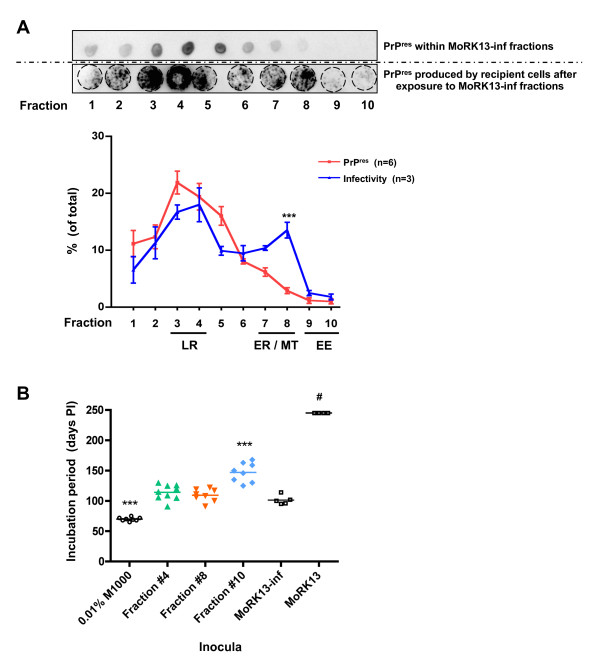
**Disparity in subcellular fraction PrP^res ^levels and relative infectivity *in vitro *and *in vivo***. (A) Relative amounts of PrP^res ^contained within MoRK13-inf fractions (dot blot - top panel, also Figure 2; red line) plotted against relative infectivity of each fraction (PrP^res ^produced by recipient MoRK13 - bottom panel, also Figure 3; blue line). There is a highly significant separation of relative PrP^res ^and infectivity levels observed in fraction #8; statistical analysis was by two way ANOVA; *** p < 0.001. Fractions enriched in lipid raft (LR), endoplasmic reticulum (ER), mitochondrial (MT) and early endosome (EE) marker proteins are highlighted. (B) Mean survival (horizontal bar) of Tga20 mice inoculated with 0.01% (w/v in PBS) M1000 brain homogenate, whole cell lysate from MoRK13 and MoRK13-inf cells, and selected subcellular fractions from MoRK13-inf. Each data point represents the number of days post-inoculation (PI) that a terminally ill mouse was sacrificed, with the exception of Tga20 mice inoculated with MoRK13 cell lysate (#) which were symptom free when culled at 245 days PI. Mice inoculated with 0.01% (w/v) M1000 brain homogenate had a significantly shorter incubation period than those exposed to all other inocula. Mice inoculated with fraction #10 had a significantly longer incubation period compared to mice exposed to all other infectious inocula. Statistical analysis was by one way ANOVA; *** p < 0.001.

Correlation of the percentage of PrP^res ^present in each fraction with the level of *in vitro *infectivity produced by each fraction (Figure 4A) revealed a discrepancy in the ER/MT enriched fractions. The relative levels of PrP^res ^present in the lipid raft enriched fractions (#3 & #4) and the early endosome enriched fractions (#9 and #10) correspond with the relative levels of PrP^res ^produced by the recipient MoRK13 cells. In contrast, the ER/MT enriched fractions (#7 and #8) that display low detectable PrP^res ^levels, contained high infectivity levels similar to the lipid raft enriched fractions, with the discrepancy at fraction #8 highly significant (p < 0.001; two way ANOVA). Therefore, there was a reproducible and significant divergence between PrP^res ^and infectivity levels within ER/MT marker enriched fractions.

To confirm genuine prion infectivity, selected fractions were bioassayed in Tga20 PrP^C ^over-expressing mice. The fractions were chosen to provide a range of PrP^res ^and infectivity level combinations; ie high PrP^res ^and infectivity levels (fraction #4), low PrP^res ^and infectivity levels (fraction #10), and low PrP^res ^but high infectivity levels (fraction #8). Controls included inoculating mice with whole cell lysate from naive MoRK13 and MoRK13-inf cells, as well as M1000 brain homogenate, in order to make comparisons with the original prion strain. Figure 4B depicts the incubation periods for the selected fractions and control mice. Mice exposed to uninfected MoRK13 cell lysate were symptom free at 245 days post-inoculation. Mice inoculated with 0.01% M1000 brain homogenate had a significantly shorter incubation period than mice inoculated with MoRK13-inf whole cell lysate. Importantly, concordant with the *in vitro *cell culture transmissions, mice inoculated with fractions #4 and #8 had indistinguishable incubation periods, despite the significantly different PrP^res ^levels contained within these fractions. Mice inoculated with fraction #10 had significantly longer incubation periods than mice infected with the other two fractions, again concordant with the relative infectivity levels determined by the cell culture transmissions. For illustrative purposes, this 35 day extension in incubation period approximates a 3 log reduction of infectious titre when modelled on a time interval assay developed in Tga20 mice inoculated with M1000 brain homogenate derived prions (Additional file 4: Figure S4). In summary, the *in vivo *findings faithfully recapitulate and validate the MoRK13 *in vitro *model.

### Infectious prions from different subcellular fractions do not induce unique disease *in vivo*

**Figure 5:**
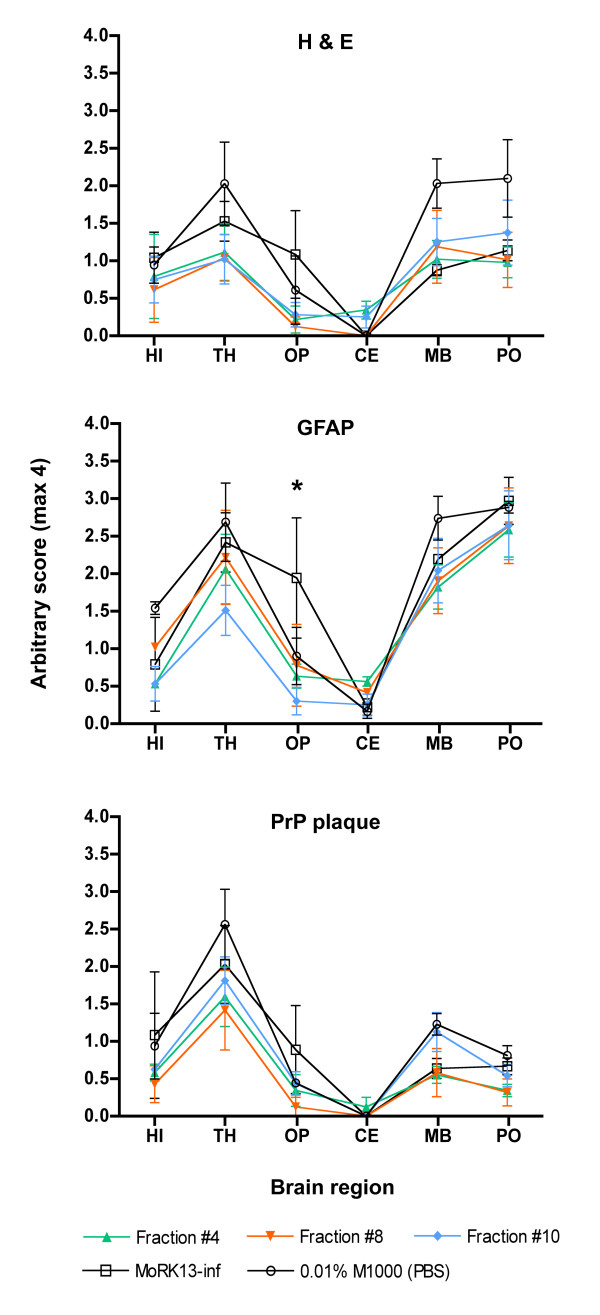
**Lesion profiles from Tga20 mice intracerebrally inoculated with M1000, MoRK13 and MoRK13-inf whole cell lysates and selected MoRK13-inf fractions**. Lesion profiles generated by quantification of the degree of vacuolation, reactive astrocytosis and PrP plaques in each brain region (HI - hippocampus, TH - thalamus, OP - occipital pole, CE - cerebellum, MB - midbrain, PO - pons) in Tga20 mice inoculated as indicated. n = 3 or 4 for all brain regions except in MoRK13-inf whole lysate HI H&E and 0.01% M1000 HI PrP plaque deposition, where n = 2. Statistical analysis comparing lesion profiles was by two way ANOVA. The only significant difference was comparing MoRK13-inf and Fraction #10 in OP region (*p < 0.05).

Neuropathologically, prion diseases are characterised by the presence of neuronal loss, spongiform change, proliferation of astrocytes and extracellular PrP deposits [37]. Further, for different prion strains the topographical distribution of neuropathological changes is distinctive, creating characteristic lesion profiles [38,39]. All diseased mice displayed symptoms typical of M1000 prion infection and brains were harvested to assess neuropathological changes. As expected, control mice inoculated with MoRK13 whole cell lysate showed no neuropathological abnormalities (Additional file 5: Figure S5). Figure 5 displays the quantification of the neuropathological features mentioned above within different brain regions, clearly indicating that each inoculum produced very similar lesion profiles (for representative photomicrographs of the histological and immunohistochemical findings see Additional file 5: Figure S5). This distribution pattern of the pathological features, with predominant involvement of the thalamus, midbrain and pons, and minimal involvement of the cerebellum is consistent with previous observations of the M1000 strain [40,41]. The only significant difference detected in lesion profiles was between MoRK13-inf whole cell lysate and fraction #10 inocula, with the former having significantly more reactive astrocytes in the occipital cortical region. The explanation for this is uncertain, but may suggest an additive effect of the individual fractions, which contribute to the whole-cell lysate profile. Different prion strains often have distinctive PrP^res ^glycoform ratios and mobilities on western blot analysis [42-46]. To further evaluate the possibility of different MoRK13-inf subcellular fractions associating with distinct prion strains, the brain PrP^res ^profiles from Tga20 mice inoculated with selected fractions were examined by western blot. Similar to the lesion profiling, there were no significant differences in PrP^res ^patterns when comparing MoRK13-inf whole cell lysate with each of the selected fractions, in keeping with them all being the same prion strain (Additional file 6: Figure S6).

### The efficiency of M1000 prion transmission is not enhanced by exogenous fraction specific co-factors *in vitro *or *in vivo*

**Figure 6:**
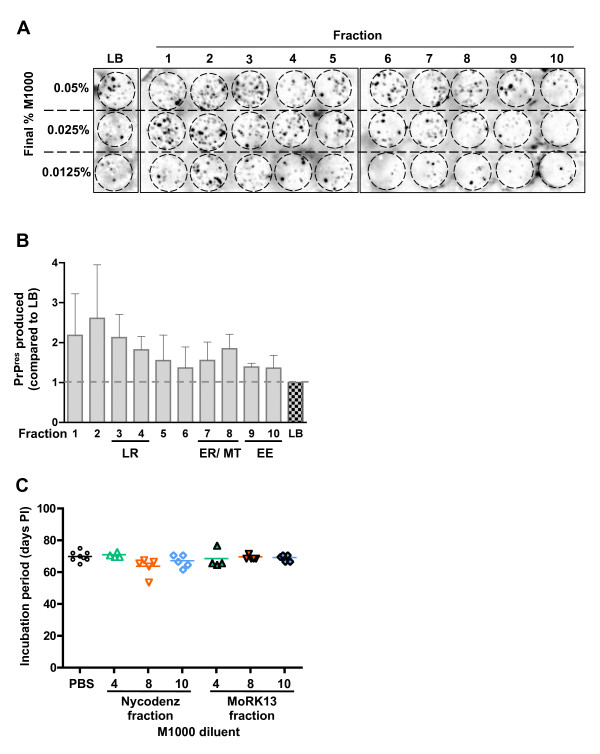
**Exogenous cellular co-factors do not modify the efficiency of M1000 infection *in vitro *or *in vivo***. (A) Representative cell blot showing PrP^res ^levels produced by recipient MoRK13 cells exposed to M1000 brain homogenate diluted to three different final concentrations (as indicated) with either 1:4 lysis buffer (LB):medium or 1:4 MoRK13 fraction:medium mix. (B) Quantification of PrP^res ^produced by MoRK13 cells exposed to M1000 brain homogenate diluted in subcellular fraction relative to the equivalent % M1000 brain homogenate diluted in lysis buffer; data is from M1000 spiked into three independent fractionations of MoRK13. For quantification purposes, the three different % M1000 spikes into the same MoRK13 fractionated lysate were considered a triplicate of the same experiment. Fractions enriched in lipid raft (LR), endoplasmic reticulum (ER), mitochondrial (MT) and early endosome (EE) marker proteins are highlighted. Analysis of relative levels of PrP^res ^produced after infection was by one-way ANOVA with Tukey's multiple comparison test; no significant differences found comparing subcellular fractions. (C) Survival of Tga20 mice inoculated with M1000 brain homogenate diluted to 0.01% with either PBS, selected fractions from an empty Nycodenz gradient, or selected subcellular fractions from MoRK13 cells. Each data point represents the number of days post-inoculation (PI) which mice were sacrificed because of terminal prion disease. Statistical analysis was by one way ANOVA, with no significant differences in survival seen between any inocula.

In order to explore whether subcellular fraction specific co-factors might contribute to the observed transmission efficiency spectrum, M1000 brain homogenate was spiked into fractions derived from uninfected MoRK13 or vecRK13 cells, prior to use as inocula *in vitro*. Figure 6A shows that even at the lowest dilutions of M1000 in MoRK13 fractions, PrP^res ^was produced by recipient MoRK13 cells. However, quantification (Figure 6B) shows there were no significant differences in amounts of PrP^res ^produced when comparing the various MoRK13 subcellular fraction diluents. Notably, there was a general trend for MoRK13 subcellular fractions to enhance M1000 transmission efficiency to MoRK13 cells compared to lysis buffer alone. A similar result was observed using fractions obtained from vecRK13 cells (Additional file 7: Figure S7), suggesting this trend is not PrP^C ^specific or dependent.

To further evaluate the possible contribution of cellular co-factors on the efficiency of prion infection, M1000 brain homogenate was diluted in PBS or selected subcellular fractions from uninfected MoRK13 cells (fractions #4, #8 and #10), and bioassayed in Tga20 indicator mice. M1000 was also diluted in 'empty' Nycodenz fractions (#4, #8 and #10), in order to control for any affect the Nycodenz gradient material itself may have on incubation period or the neuropathology. Neither the exogenous cellular co-factors in the selected fractions, nor Nycodenz alone, had any significant affect on the incubation time (Figure 6C) or overtly affected the neuropathology (Addtional file 8: Figure S8) of M1000 in Tga20 mice.

### Discrepancies between PrP^res ^and transmission efficiency are not due to protease-sensitive prions, and only in lipid raft enriched fractions is infectivity enhanced by sonication

**Figure 7:**
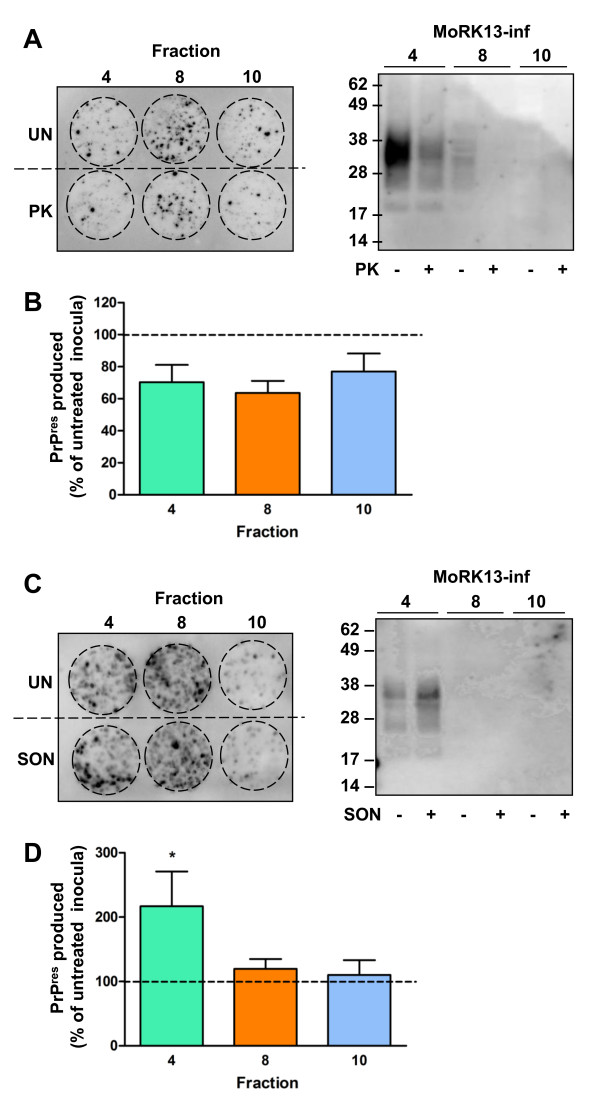
**Sonication but not PK treatment alters efficiency of *in vitro *infection with fraction #4 only**. (A) Representative cell blot (left panel) of PrP^res ^produced by MoRK13 cells exposed to selected MoRK13-inf fractions which were PK digested (PK) or left untreated (UN) and western blot (right panel) of MoRK13-inf inocula before and after PK treatment. (B) Quantification of cell blot PrP^res ^levels (n = 3 independent experiments), with PrP^res ^produced after infection with PK treated inocula expressed relative to untreated MoRK13-inf fraction inocula. (C) Representative cell blot (left panel) of PrP^res ^produced by MoRK13 cells exposed to selected MoRK13-inf fractions which were sonicated (SON) or left untreated (UN) and western blot (right panel) of MoRK13-inf fraction inocula before and after sonication. (D) Quantification of cell blot PrP^res ^levels expressed relative to untreated MoRK13-inf fraction inocula (n = 3 independent experiments). Note, levels of total PrP in fractions #8 and #10 are at or below the limits of detection by standard immunoblotting. Comparison of relative PrP^res ^levels by one way ANOVA and Dunnett's post-test for specific comparisons to a control (ie untreated fraction) found no significant differences in any PK treated inocula, and only sonicated MoRK13-inf fraction #4 was significantly different compared to its untreated control, *p < 0.05.

There is experimental evidence that some disease associated [47-50], and synthetic [51] prions, may be protease sensitive. To test whether the infectious prions within the MoRK13-inf fractions may differentially contain significant amounts of protease sensitive species, selected fractions were digested mildly with PK prior to using them as a source of infectivity. There were no significant differences in PrP^res ^levels produced by MoRK13 infected with PK digested fractions #4, #8 or #10 compared to cells infected with the untreated fraction counterpart (Figure 7A, B). However there was a modest general trend towards reduced PrP^res ^production by recipient cells after PK treatment of each fraction inoculum.

Evidence suggests misfolded prion protein aggregate size correlates with efficiency of conversion or prion infection [30,31]. As a consequence, *in vitro *conversion assays such as PMCA may be most efficient when incorporating sonication steps [52,53]. To assess this possibility, selected fractions (#4, #8 and #10) were subjected to sonication (equivalent to one 'round' of PMCA) prior to using them as a source of infectivity in the MoRK13 cell culture model. As demonstrated by the representative cell blot in Figure 7C, quantified in Figure 7D, only subcellular fraction #4 had significantly increased levels of infectivity compared to its untreated fraction.

## Discussion

The protein-only hypothesis states that misfolded conformers of the normal cellular prion protein are the principal component of the agent responsible for transmitting prion disease [1]. However, previously observed examples of high infectivity titres associated with very low or undetectable PrP^res^, the commonly utilized surrogate prion marker, suggest a poorly understood spectrum of infectious prions. Assessing the subcellular environment of the most infectious prions may provide information about optimal pH or metal content conditions, implicate membrane domains, or subcellular co-factors involved in localisation of highly efficient prions. Investigation of the subcellular distribution of prion infectivity and corresponding PrP^res ^levels has been previously reported, albeit to a limited extent. However, absent in prior studies were attempts to explore what determines the intracellular topographical diversity of prions or the molecular basis of any observed discrepancies in PrP^res ^and infectivity levels.

The present study clearly indicates that not all prion infectivity is associated with lipid rafts, although the significance of the lipid raft microenvironment in PrP^C ^misfolding and prion conversion is yet to be resolved, with experimental evidence both for and against lipid raft localisation as an optimal site (reviewed in [16]). In complete agreement with the protein-only hypothesis, lipid raft and EE marker associated infectivity and PrP^res ^were shown to correlate in the MoRK13-inf model. In contrast however, ER/MT marker enriched fractions contained much greater infectivity when reported to relative PrP^res ^content. That lipid raft and ER/MT enriched fractions contain the same infectivity levels indicates some biological redundancy or relative inefficiency of the lipid raft localised prions, and/or higher efficiency of ER/MT localised prions, prompting further investigation.

Cell-free conversion studies have shown there is a role for cellular co-factors, such as nucleic acid or other polyanionic molecules, in the efficiency of prion conversion and propagation [21-23,29,54]. Perhaps militating against a prominent role of specific co-factors contributing to the transmission efficiency of the MoRK13-inf subcellular fractions, infectivity of brain derived M1000 prions was not significantly differentially enhanced by dilution across various subcellular fractions. However, an explanation for this result is that the transmissible prions within M1000 brain homogenate were already largely in an optimal state, pre-formed and associated with the necessary co-factors required for infection. Therefore additional exogenous co-factors supplied via the MoRK13 or vecRK13 subcellular fractions were somewhat superfluous. Alternatively, the fractionation procedure itself may have inactivated any critical co-factors, such that they were not able to significantly enhance the infectivity of M1000 brain homogenate. In fact, there was a clear trend for increased PrP^res ^propagation by recipient cells after infection with M1000 diluted in subcellular fractions compared to the lysis buffer only control, independent of whether the fractions contained PrP^C^. This may indicate that incompletely defined but relatively ubiquitous 'cellular co-factors' contribute to the efficiency of *in vitro *prion transmission, which would be consistent with previous studies.

Numerous prion strains exist, evident in both naturally occurring human [55,56] and animal [38,57-60] prion disease, as well as those adapted to laboratory based animal models. One hypothesis for what determines different strains is the tertiary structure of the prion conformer, possibly affected by metals, co-factors or binding partners [61]. It is also believed that prions may adopt various stable tertiary conformations, and there is evidence of simultaneous propagation of more than one prion strain within the brain [55,62,63]. Furthermore, super-infection experiments indicate that the more infectious strain will predominate and determine disease expression [64-66]. Prion strains can be classified by their distinctive neuropathological lesion profiles, incubation periods, PrP^res ^glycosylation patterns and electrophoretic mobilities [39,42,43,46]. As RK13 cells are capable of supporting and maintaining propagation of many prion strains [67], and each fraction represented different subcellular localisations and potential binding partners, we explored the possibility that MoRK13-inf fractions contained structurally distinct prions of variable transmission efficiency. However histological and western blot analyses failed to detect any evidence of a subcellular divergence of prion strains, strongly militating against this as the explanation for the apparent increased relative infectivity in the ER/MT marker enriched fractions.

Previous experiments have shown that PrP^res ^aggregate size affects the efficiency of conversion and prion infection, perhaps through effects on optimising the available templating surface [30,31], with oligomers of five or fewer PrP^res ^molecules and larger fibrillar aggregates of PrP^res ^far less efficient than non-fibrillar particles of 14-28 molecules [30]. There is also experimental evidence that a proportion of disease associated prions are protease sensitive [47-49], which may form low molecular weight aggregates [47,49]. The results presented herein show that only a minor proportion of prion infectivity within MoRK13-inf fractions is protease sensitive, and is unlikely to account for the disconnect observed between PrP^res ^levels and infectivity in the ER/MT and lipid raft enriched fractions. Rather, the sonication results are in keeping with a greater proportion of multimeric assemblies, fibrils or aggregated species of prions existing in the lipid raft compared to ER/MT marker enriched fractions, with sonication increasing the number of replication-competent prion oligomeric strand ends which are then more efficient at transmission and inducing prion propagation. Recent publications provide credence to this hypothesis [68,69], with direct visualisation of the fragmentation of recombinant PrP after sonication. However, the current study does not exclude any positive effect that sonication may have had on other cellular components contained within the fraction mileu or interactions between the prion protein and other molecules. In fact another recent publication [70] found that sonication also fragments purified liver RNA, to a size that has previously been shown to stimulate prion conversion in PMCA assays. However the RNA sonication produced optimal (sized) RNA after approximately 8 cycles, whereas our sonication experiment was equivalent to one cycle, giving some support to the plausibility of our former hypothesis. Ongoing studies, including the utilisation of techniques such as the conformation dependent immunoassay (CDI) to measure prions in the fractions [71], and sophisticated size fractionation techniques, will help clarify the exact biophysical nature of the variably efficient prion species in the lipid raft and ER/MT enriched fractions.

The association of prion infectivity with MT and ER has been previously investigated with conflicting results. One study showed that purified mitochondria and mitoplast fractions from scrapie infected hamster brain contained infectivity titres equivalent to those determined for crude brain homogenate, yet mitoplast fractions were not associated with detectable levels of PrP [72], in keeping with the findings of the present study. These results, which suggested an association of high levels of scrapie infectivity with the inner mitochondrial membrane or mitochondrial matrix, are broadly consistent with the characteristics of MoRK13-inf ER/MT enriched fractions, which co-localised with the mitochondrial membrane marker Bcl-2 [73]. An integral and unique (to mitochondria) lipid component of the inner mitochondrial membrane is cardiolipin, a form of dimeric phosphatidylglycerol [74]. Interestingly, utilizing serial PMCA, researchers have recently been able to produce protease resistant, infectious prions from recombinant PrP mixed with RNA and the synthetic phosphatidylglycerol, POPG (1-palmitoyl-2-oleoylphosphatidylglycerol) [75]. The authors state that the POPG and RNA additives to their PMCA may be mimicking factors which facilitate the conversion process *in vivo*, which is entirely consistent with the results presented here implicating mitochondrial component enriched fractions as containing highly efficient infectious prions.

Somewhat incongruent with these observations, a much earlier study examined the infectivity of scrapie within membrane fractions and found that brain derived purified mitochondrial fractions were associated with very little infectivity [76]. Nevertheless, similar to our results, Millson and colleagues [76] did find both brain and spleen derived subcellular fractions containing elevated enzyme activities usually associated with the ER and plasma membrane were associated with high scrapie infectivity. Conversely, Alais and colleagues [77] found fractions enriched in the ER marker Bip harboured no infectivity, despite containing moderate levels of PrP^res^. This result, whilst presenting another example of discrepancy between PrP^res ^levels and infectivity, clearly contrasts with what was observed in the MoRK13-inf fractions, perhaps reflecting the different methods and prion strain-cell model employed.

## Conclusions

Through the use of both *in vitro *and *in vivo *transmission studies, we have corroborated previously reported discrepancies between absolute PrP^res ^levels and infectivity and provided insight into the basis of this phenomenon. Through subcellular separation of infectivity, our data indicates that a substantial amount of infectivity is contained outside of buoyant lipid raft fractions and importantly showed that the most transmission efficient prions per detectable PrP^res ^unit were associated with either ER and/or MT membranes or proteins. We established that the high transmission efficiency shown by the ER/MT containing fractions was not due to the simultaneous separation of a more potent prion strain. As critical co-factor enrichment could not be completely excluded, it remains to be determined whether cellular microenvironments directly but variably contribute to the transmission efficiency of resident prions, or only passively serve as sequestration sites for the different prion species. Overall the current study broadly aligns with the notion that rather than absolute levels of PrP^res^, intrinsic prion properties may dictate or be dictated by the ultimate subcellular localisation of infectious prions, with transmission efficiency likely correlating best with optimal prion oligomeric state for template directed conversion. Importantly, through the development and validation of a tractable model, further detailed exploration of these fundamental aspects of prion biology, including assessment of other cell line-prion strain combinations to determine the breadth of applicability of our observations, can be undertaken.

## Methods

### Cell culture

Rabbit kidney epithelial cells which have no detectable endogenous PrP^C ^protein, were stably transfected to over-express murine PrP^C ^(MoRK13) or the empty vector (vecRK13) [78] and mouse hypothalamic GT1-7H cells were maintained as described previously [79], in a humidified incubator at 37°C with 5% CO_2_.

### Subcellular fractionation

A method of non-toxic/non-detergent cell lysis and subcellular separation was necessary to allow subsequent use of fractions for infecting recipient cells or mice. Also, due to the possible involvement of lipid rafts in prion conversion, a lysis method was chosen in order to maintain lipid raft integrity and buoyancy [35], with minor modifications. Briefly two confluent T175 cm^2 ^flasks (approximately 4 x10^7 ^cells) were washed twice with 20 mls ice cold lysis buffer (20 mM Tris-Cl pH 7.8, 250 mM sucrose, 1 μM CaCl_2_, 1 μM MgCl_2_) and harvested by scraping into a further 20 ml of lysis buffer and pelleting at 700 × g for 3 minutes. The cell pellet was re-suspended in 500 μl cold lysis buffer. Cells were lysed on ice, by passing the cell suspension through a 22 g needle exactly twenty times, and the crude lysate was centrifuged at 1000 × g, 10 minutes at 4°C. The post-nuclear supernatant was retained on ice and the extraction repeated on the pellet. The lysate was then assayed for total protein content by performing a bicinchoninic acid (BCA) assay (Pierce, Thermo Scientific, Scoresby, VIC, AUS) as per the manufacturer's instructions, and adjusted with lysis buffer to 1.8 mg/ml. The Nycodenz (HistoDenz™, Sigma-Aldrich, Castle Hill, NSW, AUS) density gradient fractionation method was adapted from a published protocol [80], to suit a Beckman Optima Max-E Benchtop Ultracentrifuge and MLS-50 rotor. Nycodenz solutions were prepared in TNE (25 mM Tris-Cl pH 7.5, 150 mM NaCl, 5 mM EDTA) and an 8-35% linear step Nycodenz gradient was poured (400 μl of each of 8%, 12%, 15%, 18%, 20%, 22.5% and 25%) with 1.1 ml of a 35% Nycodenz cushion consisting of equal parts ice cold 70% Nycodenz and cell lysate (1 mg total protein) pipetted to the bottom of the gradient. In some cases, an 'empty Nycodenz' gradient was poured, whereby the 35% Nycodenz/lysate cushion mixture was substituted for 35% Nycodenz alone. Nycodenz gradients were centrifuged at 200,000 × g (average) for 342 minutes at 4°C. Following centrifugation, 10 equal volume fractions of 390 μl were collected and stored at -80°C or kept on ice for immediate use.

### Prion strain and cell infections

The M1000 prion strain used in this study was derived from a well characterised stock of pooled mouse brain homogenate [81]. Recipient MoRK13 or GT1-7H cells were infected using an overlay technique as described previously [79]. For comparisons of cell lysate and brain homogenate M1000 infectivity, cell lysates were prepared by harvesting and lysing in sterile phosphate buffered saline (PBS; Invitrogen, Mulgrave, VIC, AUS) by three cycles of freezing (10 minutes at -80°C) and thawing (3 minutes at 37°C), and centrifugation at 1000 × g for 3 minutes at 4°C to obtain a post-nuclear supernatant. The total protein content of the PBS supernatant and M1000 brain homogenate were determined by BCA assay, and the lysates and homogenate were balanced to the same protein concentration with PBS. Following this 100 μl of lysate or homogenate was mixed with 400 μl of complete medium and this was used to infect recipient cells. For fraction infections 100 μl fraction ('neat') or, where indicated, fraction which had been serially diluted in medium, was mixed with 400 μl complete medium, and used to infect recipient MoRK13 cells. For 'spiking' experiments, M1000 brain homogenate was diluted in 400 μl media and mixed with 100 μl MoRK13 or vecRK13 fraction (or as a control the lysis buffer used to prepare cells prior to fractionation) to give a final concentration of 0.05%, 0.025% and 0.0125% M1000, prior to being used to infect recipient MoRK13 cells.

### Subcellular fraction pre-treatments

For PK digestion, 100 μl of a fraction was treated with a final concentration of 1 μg/ml PK for 8 hours at 37°C, conditions found to reduce PrP^C ^by approximately 80% when tested on control fractions (data not shown). For sonication pre-treatment, 100 μl of a fraction in a 1.5 ml microfuge tube was subjected to 60 seconds at amplitude 70 in a S4000 sonicator (Misonix, Farmingdale, NY, USA) with microplate horn adapter in 300 mL water maintained at 37°C. The PK digested or sonicated fractions (and untreated controls) were mixed with 400 μl of complete medium and used for *in vitro *infections as described above.

### Immunoblotting

For determination of subcellular localisation of PrP and other proteins, fractions were mixed with 4X sample buffer and subject to PAGE (using either 4-20% or 10-20% Tris-glycine SDS or 4-12% Bis-tris NuPAGE pre-cast gels (Invitrogen), depending on the size of the proteins to be detected, and then transferred to PVDF membrane for western blotting of PrP as described previously [79] and organelle marker proteins using antibody dilutions as outlined in the manufacturer's instructions (BD Pharmingen™ Organelle Sampler Kit, BD Biosciences, North Ryde, NSW, AUS). For detection of the ganglioside GM_1 _(lipid raft marker), 3 μl of each fraction was spotted onto nitrocellulose membrane, and allowed to dry for 20 minutes at 37°C. The membrane was blocked for a minimum of 1 hour in 5% skim milk powder in PBS containing 0.05% (v/v) Tween-20 (PBST) and then incubated in 1:100,000 cholera toxin B subunit (CTB)-horseradish peroxidise (HRP) conjugate (Sigma, stock concentration 0.45 mg/ml CTB and 1 mg/ml HRP in H_2_O) solution in block for 1.5 hours at room temperature prior to chemiluminescent detection (ECL Plus, GE Healthcare, Rydalmere, NSW, AUS). For PrP^res ^detection, fractions, cell lysates or brain homogenate were digested with a final concentration of 50 μg/ml PK, 1 hour at 37°C before SDS-PAGE and western blotting. Dot blots of fractions were also carried out for PrP^res ^detection, whereby 5 μl of fraction was spotted onto nitrocellulose membrane, which was dried 30-60 minutes at 37°C and then treated in exactly the same manner as the cell blot assay nitrocellulose membrane. Cell blots for the detection of PrP^res ^in recipient cells, at four passages (P4) post-infection were carried out as described previously [79]. All immunoblotting (western blots and cell blots) for PrP species used the monoclonal antibody ICSM18, (D-Gen, London, UK). All chemiluminescent images were captured by a Fujifilm LAS-3000 (Berthold Australia, Bundoora, VIC, AUS).

### *In vivo *prion transmissions

All animal experiments were carried out in strict accordance with the 'Australian Code of Practice for the Care and Use of Animals for Scientific Purposes (NHMRC)', with approval from the University of Melbourne Animal Ethics Committee (AEC #04154). Tga20 PrP^C ^over-expressing mice [82] were anesthetised using methoxyfluorane and inoculated intracerebrally with 30 μl of 0.01% M1000 brain homogenate diluted in PBS or the appropriate fractions as indicated, or with 30 μl of 'neat' fraction or whole cell PBS lysate. Mice were provided food and water *ad libitum *and housed following routine animal husbandry practices. Mice were examined daily for symptoms of prion disease. Once mice developed persisting features of advanced prion disease, including impaired righting reflexes, hunched posture and hind limb paresis, they were culled by cervical dislocation under anaesthesia and the number of days post-inoculation was recorded. Brains were removed and sagittally hemi-sectioned, with half the brain fixed and stained to allow scoring of vacuolation, astrocytic gliosis and PrP deposition as described previously [40], and the other half made to 10% (w/v) homogenates in PBS, with homogenates stored at -80°C until required. Neuropathological scoring was performed on two separate occasions, blinded as to the inoculum group, to provide a semi-quantitative comparison of lesion profiles between the groups of animals. Stained sections were visualised using a Zeiss Axioskop 50 microscope with images captured using a Zeiss AxioCam HRC camera (Carl Zeiss, North Ryde, NSW, AUS).

### Densitometry and statistical analysis

All densitometric analyses used the public domain ImageJ software (National Institutes of Health, USA). For determining relative levels of PrP^res ^in fractions, each fraction PrP^res ^dot blot signal intensity was measured, with the sum of the 10 individual PrP^res ^levels providing the 'total PrP^res^'; each individual fraction was expressed as a percentage of the total PrP^res^. For determining relative levels of infectivity contained within MoRK13-infectious fractions, PrP^res ^produced by MoRK13 cells exposed to each 'neat' fraction was measured at passage 4 (P4) post-exposure by cell blot signal intensity, with the sum of the 10 individual PrP^res ^levels providing 'total PrP^res^'. Once again, each individual fraction was expressed as a percentage of the calculated total PrP^res^. All statistical analyses were performed in GraphPad Prism 4, with one way ANOVA and Tukey's multiple comparisons or two way ANOVA and Bonferonni post-tests used as indicated, unless stated otherwise.

## Abbreviations

BCA: Bicinchoninic acid; CDI: Conformation dependent immunoassay; CTB: Cholera toxin B subunit; EE: Early endosome; ER: Endoplasmic reticulum; MT: Mitochondria; PBS: Phosphate buffered saline; PK: Proteinase K; PMCA: Protein misfolding cyclic amplification; POPG: 1-palmitoyl-2-oleoylphosphatidylglycerol; PrP: Prion protein; PrP^C^: Cellular prion protein; PrP^res^: Protease resistant prion protein.

## Competing interests

The authors declare that they have no competing interests.

## Authors' contributions

VL performed all experiments. VL, CLH and SJC were involved in the acquisition of data. VL, AFH, VAL and SJC contributed to experimental conception and design. VL, CLH, CLM, AFH, VAL and SJC were involved in analysis and interpretation of data and production of this manuscript. All authors read and approved the final manuscript.

## Supplementary Material

Additional file 1 **Figure S1**. Disparity in PrP^res ^levels and relative infectivity in the MoRK13-inf cell prion infection model. Representative western blot (A) and quantification (B) of PrP^res ^levels in MoRK13-inf whole cell lysates relative to M1000 brain homogenate (n = 3). Samples were balanced for total protein; 5 μg total protein was resolved in untreated (-) lanes; 50 μg total protein was proteinase K (PK) digested (+) and resolved on 4-12% Bis-tris NuPAGE gels. Very long exposures were required to visualise the PrP^res ^within cell lysates, hence the loss of distinction between M1000 PrP^res ^glycoforms. Representative cell blot (C) and quantification (D) of PrP^res ^produced by recipient MoRK13 cells infected with equivalent total protein amounts of M1000 or MoRK13-inf cell lysate (n = 3). Note infections utilised the corresponding lysate/homogenate shown in (A). NBH = Balb/c normal brain homogenate. Statistical analysis by one way ANOVA; *** p < 0.001, ** p < 0.01. Click here for file

Additional file 2 **Figure S2**. Localisation of PrP, organelle and membrane markers in Nycodenz density gradient fractions of alternative cell lines. Representative immunoblots of subcellular fractions obtained from vector only transfected RK13 cells (vecRK13), GT1-7H and (M1000 infected) GT1-7H-inf, subject to SDS-PAGE and western blotting as described in the methods. CTB dot blot - 3 μl of fraction was dried onto nitrocellulose membrane, and blotted as described in the methods. LR = lipid raft; ER = endoplasmic reticulum; MT = mitochondria; EE = early endosome; CTB = cholera toxin subunit B; Flot1 = Flotillin 1; Bcl-2 = anti-apoptotic MT marker protein; Bip = ER lumen chaperone protein; EEA1 = EE antigen 1. Click here for file

Additional file 3 **Figure S3**. Localisation of PrP isoforms, organelle and membrane markers in Nycodenz density gradient fractions of M1000 brain. Representative immunoblots of subcellular fractions obtained from terminal M1000 brain. Brains were homogenised in a comparative way to the detergent-free cell lysis described in the methods, and then subject to Nycodenz gradient fractionation. Briefly, whole brains (snap frozen in liquid N_2 _and stored at -80°C) were homogenised in TNE buffer by passing the tissue through an 18 g and then a 20 g needle until an even homogenate was formed, followed by exactly 20 passes through a 22 g needle. The homogenate was centrifuged at 1000 × *g*, 10 minutes at 4°C, the post-nuclear supernatant was retained on ice and the extraction repeated on the pellet. The supernatant was then subject to Nycodenz gradient floatation, fractions were collected and analysed by SDS-PAGE and western blotting as described. PrP^res ^and CTB dot blots - 3 μl of fraction was dried onto nitrocellulose membrane, and blotted as described in the methods. CTB = cholera toxin subunit B; Flot1 = Flotillin 1; Bcl-2 = anti-apoptotic mitochondrial marker protein; Bip = ER lumen chaperone protein. Click here for file

Additional file 4 **Figure S4**. Illustration of the reduction of infectious titre in a 35-day extension of incubation period in Tga20 mice. Regression analysis (modelled on the results of an incubation time interval assay based on quantal end-point dose titration of M1000 brain homogenate in Tga20 mice) was used to plot the relationship between incubation period and titre (V.A. Lawson, unpublished data). The red and blue lines highlight an incubation period of 35 days and the corresponding titres. Click here for file

Additional file 5 **Figure S5**. Assessment of neuropathology in the brains of Tga20 mice inoculated with 0.01% (w/v) M1000 brain homogenate, MoRK13 and MoRK13-inf whole cell lysates and selected MoRK13-inf fractions. Representative photomicrographs demonstrating the degree of (A) vacuolation in hematoxylin and eosin-stained (H & E) sections, (B) astrocytic gliosis in glial fibrillary acidic protein (GFAP) stained sections, and (C) PrP plaque deposition in ICSM18 stained sections of various brain regions as indicated. Magnification = 50 × for all sections/stains except H & E stained hippocampus and occipital pole, where magnification = 100 ×. Click here for file

Additional file 6 **Figure S6**. PrP^res ^profiles in Tga20 mice inoculated with 0.01% (w/v) M1000 brain homogenate, MoRK13-inf and selected MoRK13-inf subcellular fractions. Representative western blot (A) and quantification (B) of PrP^res ^glycoform ratios in terminal mice brains after intracerebral inoculation as indicated. (A) 10 μl of PK digested (100 μg/ml final concentration PK, 1 hour at 37°C) 10% (w/v in PBS) homogenate was resolved on 12% Bis-tris NuPAGE and probed with ICSM18 primary antibody and (B) quantified using ImageJ. Di = di-glycosylated PrP^res^, mono = mono-glycosylated PrP^res^, un = unglycosylated PrP^res^. Statistical analysis by two-way ANOVA with Bonferroni post-tests; ns = no significant differences; ^#^M1000 in Balb/c mice was significantly different to all other inocula; ^0.01% M1000 in Tga20 mice was significantly different to all other inocula. Click here for file

Additional file 7 **Figure S7**. Exogenous cellular co-factors from vecRK13 do not differentially increase the efficiency of M1000 infection *in vitro*. (A) Cell blot showing PrP^res ^levels produced by recipient MoRK13 cells exposed to M1000 brain homogenate diluted to three different final concentrations (as indicated) with either 1:4 lysis buffer (LB):medium or 1:4 vecRK13 fraction:medium mix. (B) Quantification of PrP^res ^produced by MoRK13 cells exposed to M1000 brain homogenate diluted in subcellular fraction relative to the equivalent % M1000 brain homogenate diluted in lysis buffer; for quantification purposes the three different % M1000 spikes into the same vecRK13 fractionated lysate were considered a triplicate of the same experiment, with the error bars representing this intra-experiment variation, and analysis of this variation (one way ANOVA) showing no significant differences. Fractions enriched in lipid raft (LR), endoplasmic reticulum (ER), mitochondrial (MT) and early endosome (EE) marker proteins are as marked. Click here for file

Additional file 8 **Figure S8**. Lesion profiles from Tga20 mice intracerebrally inoculated with M1000 brain homogenate diluted in PBS and high density Nyocodenz with or without MoRK13 cell lysate content. Lesion profiles generated by quantification of the degree of vacuolation (H&E), reactive astrocytosis (GFAP) and PrP plaque deposition in each brain region (HI - hippocampus, TH - thalamus, OP - occipital pole, CE - cerebellum, MB - midbrain, PO - pons). n = 3 or 4 for all brain regions except in 0.01% M1000 diluted in PBS HI PrP plaque deposition, where n = 2. Statistical analysis comparing lesion profiles was by two way ANOVA. For astrocytic gliosis and PrP plaque deposition, *p < 0.05 comparing M1000 diluted in PBS and Nycodenz fraction #10 in TH region only. No other significant differences seen. Click here for file
