# Supplementary figures and images for: Prion subcellular fractionation reveals infectivity spectrum, with a high titre-low PrPres level disparity
Source: Mol Neurodegener. 2012 Apr 26;7:18. doi: 10.1186/1750-1326-7-18 (PMC3355018; doi:10.1186/1750-1326-7-18)

Fig. S1

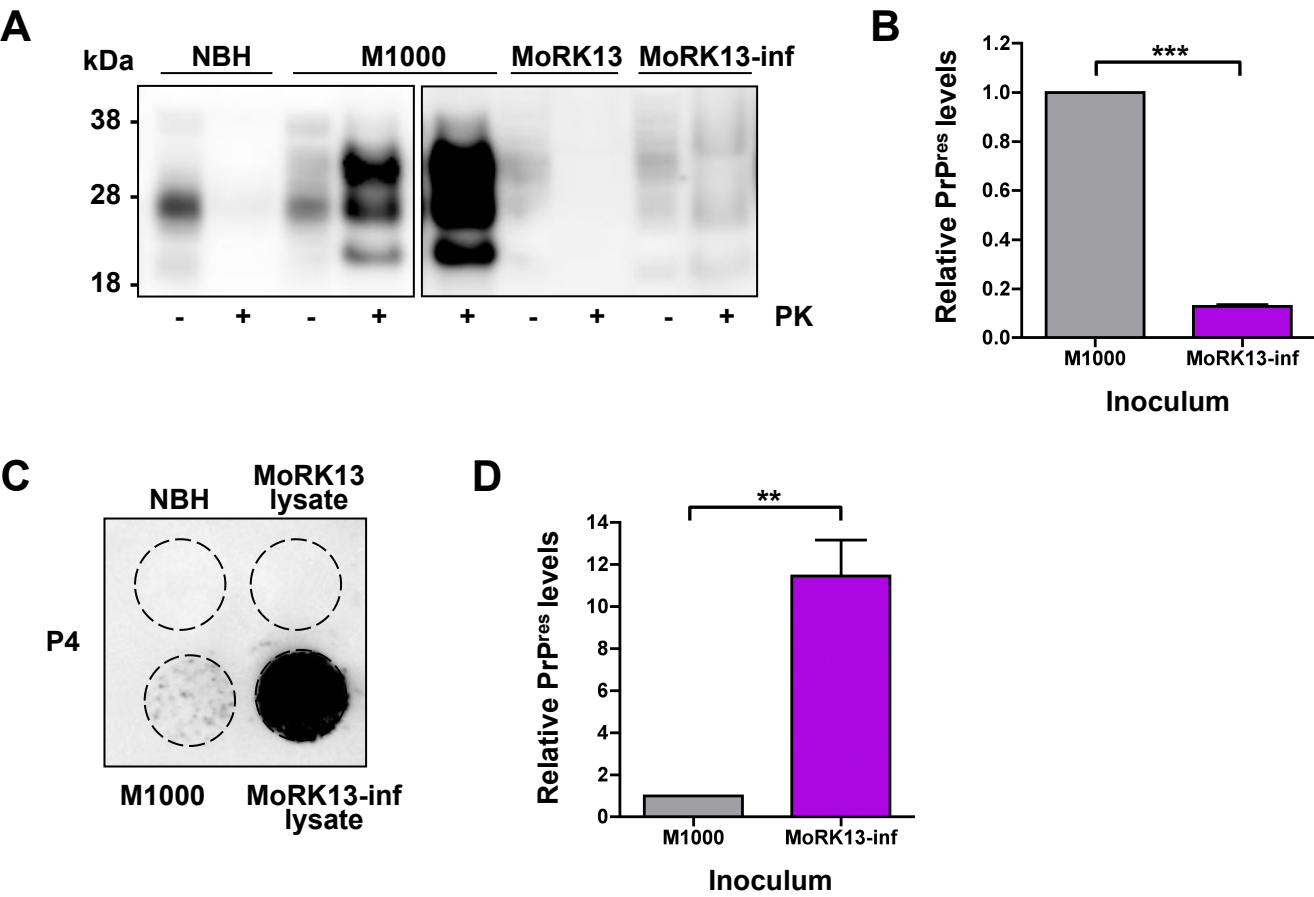

Supplement: Additional file 1 — Figure S1. Disparity in PrPres levels and relative infectivity in the MoRK13-inf cell prion infection model. Representative western blot (A) and quantification (B) of PrPres levels in MoRK13-inf whole cell lysates relative to M1000 brain homogenate (n = 3). Samples were balanced for total protein; 5 μg total protein was resolved in untreated (-) lanes; 50 μg total protein was proteinase K (PK) digested (+) and resolved on 4-12% Bis-tris NuPAGE gels. Very long exposures were required to visualise the PrPres within cell lysates, hence the loss of distinction between M1000 PrPres glycoforms. Representative cell blot (C) and quantification (D) of PrPres produced by recipient MoRK13 cells infected with equivalent total protein amounts of M1000 or MoRK13-inf cell lysate (n = 3). Note infections utilised the corresponding lysate/homogenate shown in (A). NBH = Balb/c normal brain homogenate. Statistical analysis by one way ANOVA; *** p < 0.001, ** p < 0.01. [file 1750-1326-7-18-S1.PDF]

**Fig. S2**

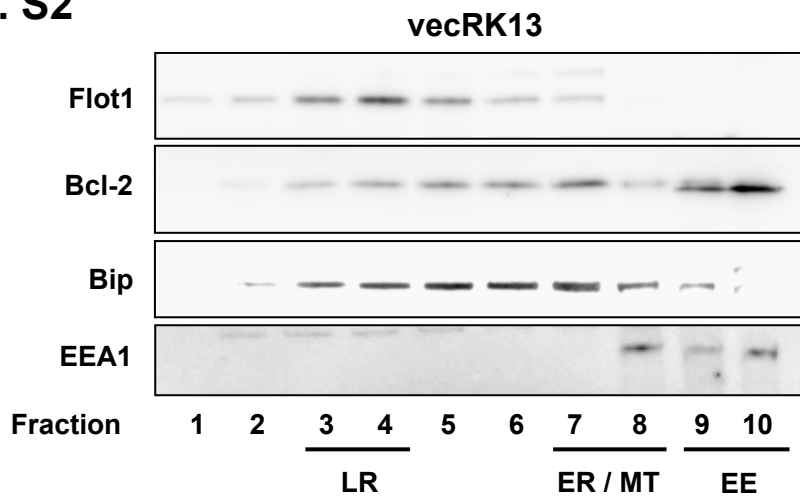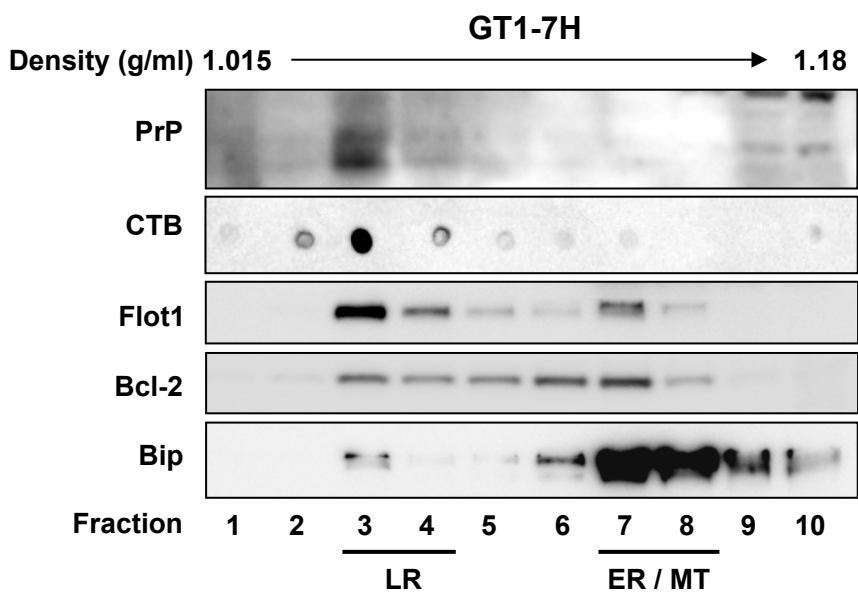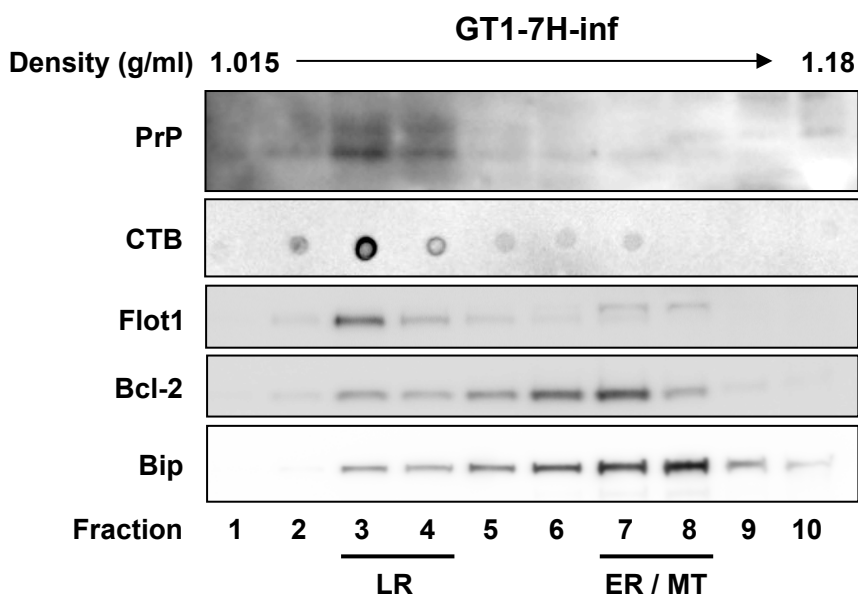

Supplement: Additional file 2 — Figure S2. Localisation of PrP, organelle and membrane markers in Nycodenz density gradient fractions of alternative cell lines. Representative immunoblots of subcellular fractions obtained from vector only transfected RK13 cells (vecRK13), GT1-7H and (M1000 infected) GT1-7H-inf, subject to SDS-PAGE and western blotting as described in the methods. CTB dot blot - 3 μl of fraction was dried onto nitrocellulose membrane, and blotted as described in the methods. LR = lipid raft; ER = endoplasmic reticulum; MT = mitochondria; EE = early endosome; CTB = cholera toxin subunit B; Flot1 = Flotillin 1; Bcl-2 = anti-apoptotic MT marker protein; Bip = ER lumen chaperone protein; EEA1 = EE antigen 1. [file 1750-1326-7-18-S2.PDF]

**Fig. S3**

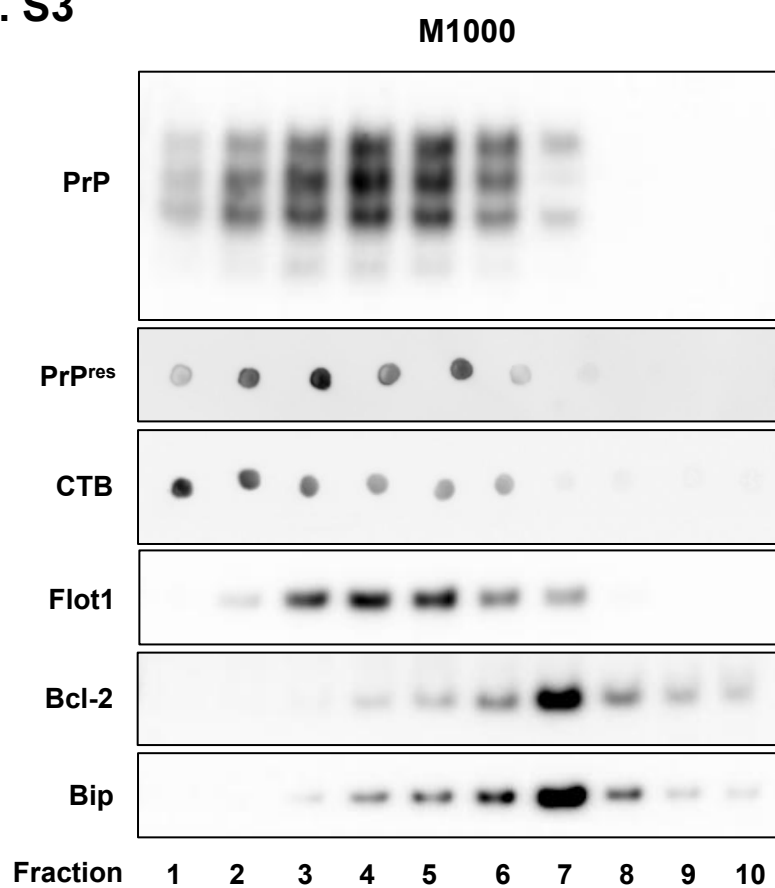

Supplement: Additional file 3 — Figure S3. Localisation of PrP isoforms, organelle and membrane markers in Nycodenz density gradient fractions of M1000 brain. Representative immunoblots of subcellular fractions obtained from terminal M1000 brain. Brains were homogenised in a comparative way to the detergent-free cell lysis described in the methods, and then subject to Nycodenz gradient fractionation. Briefly, whole brains (snap frozen in liquid N2 and stored at -80°C) were homogenised in TNE buffer by passing the tissue through an 18 g and then a 20 g needle until an even homogenate was formed, followed by exactly 20 passes through a 22 g needle. The homogenate was centrifuged at 1000 × g, 10 minutes at 4°C, the post-nuclear supernatant was retained on ice and the extraction repeated on the pellet. The supernatant was then subject to Nycodenz gradient floatation, fractions were collected and analysed by SDS-PAGE and western blotting as described. PrPres and CTB dot blots - 3 μl of fraction was dried onto nitrocellulose membrane, and blotted as described in the methods. CTB = cholera toxin subunit B; Flot1 = Flotillin 1; Bcl-2 = anti-apoptotic mitochondrial marker protein; Bip = ER lumen chaperone protein. [file 1750-1326-7-18-S3.PDF]

Fig. S4

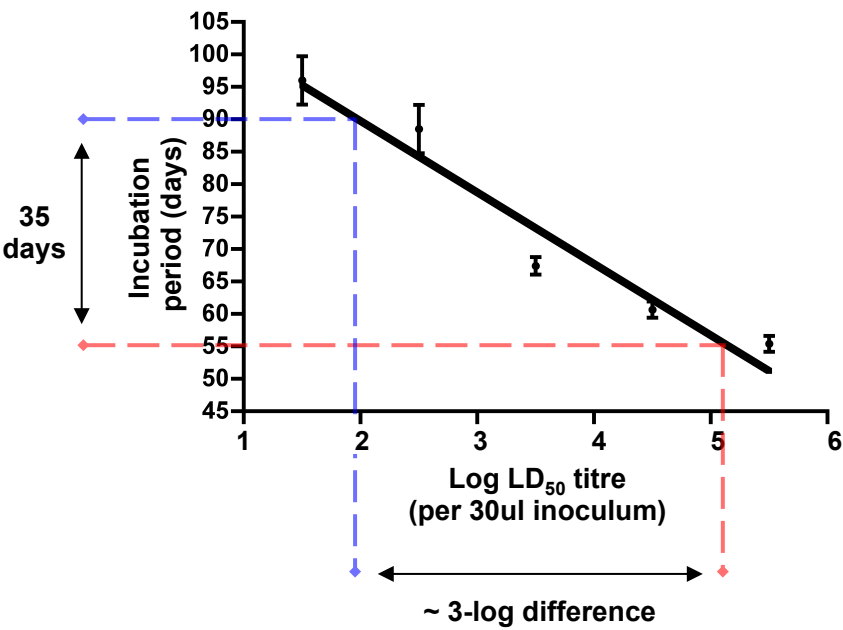

Supplement: Additional file 4 — Figure S4. Illustration of the reduction of infectious titre in a 35-day extension of incubation period in Tga20 mice. Regression analysis (modelled on the results of an incubation time interval assay based on quantal end-point dose titration of M1000 brain homogenate in Tga20 mice) was used to plot the relationship between incubation period and titre (V.A. Lawson, unpublished data). The red and blue lines highlight an incubation period of 35 days and the corresponding titres. [file 1750-1326-7-18-S4.PDF]

Fig. S5

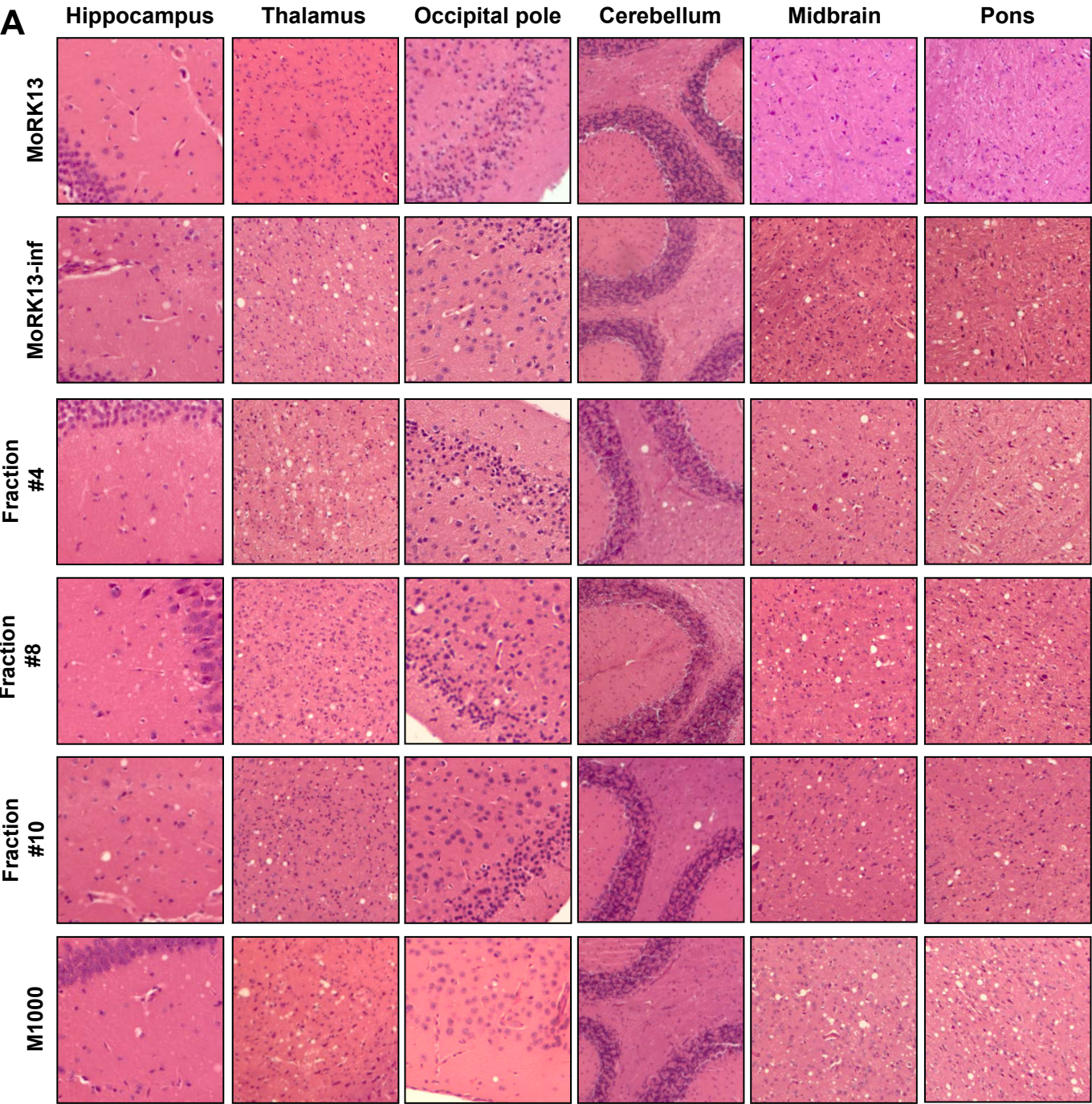

Fig. S5

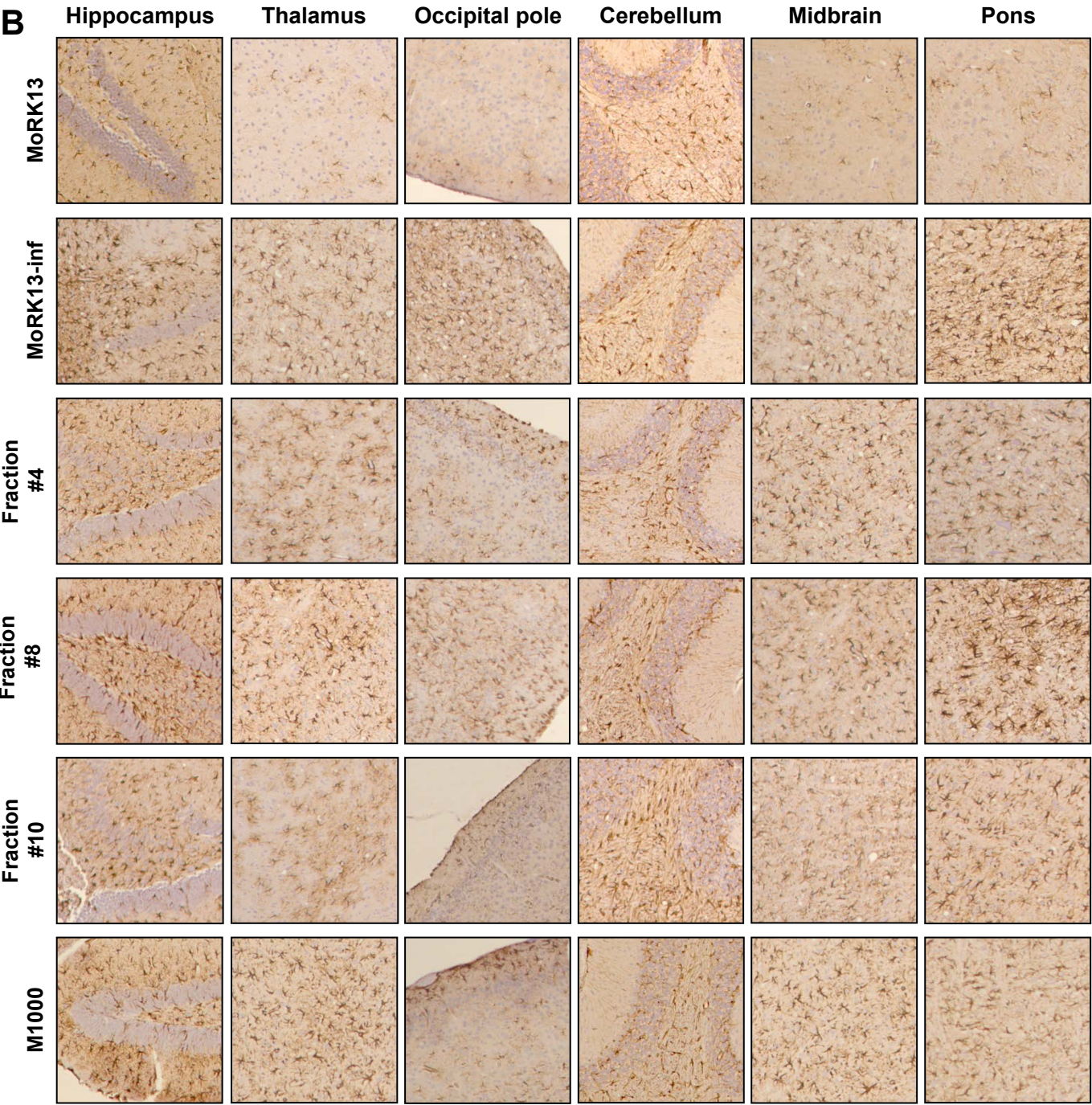

Fig. S5

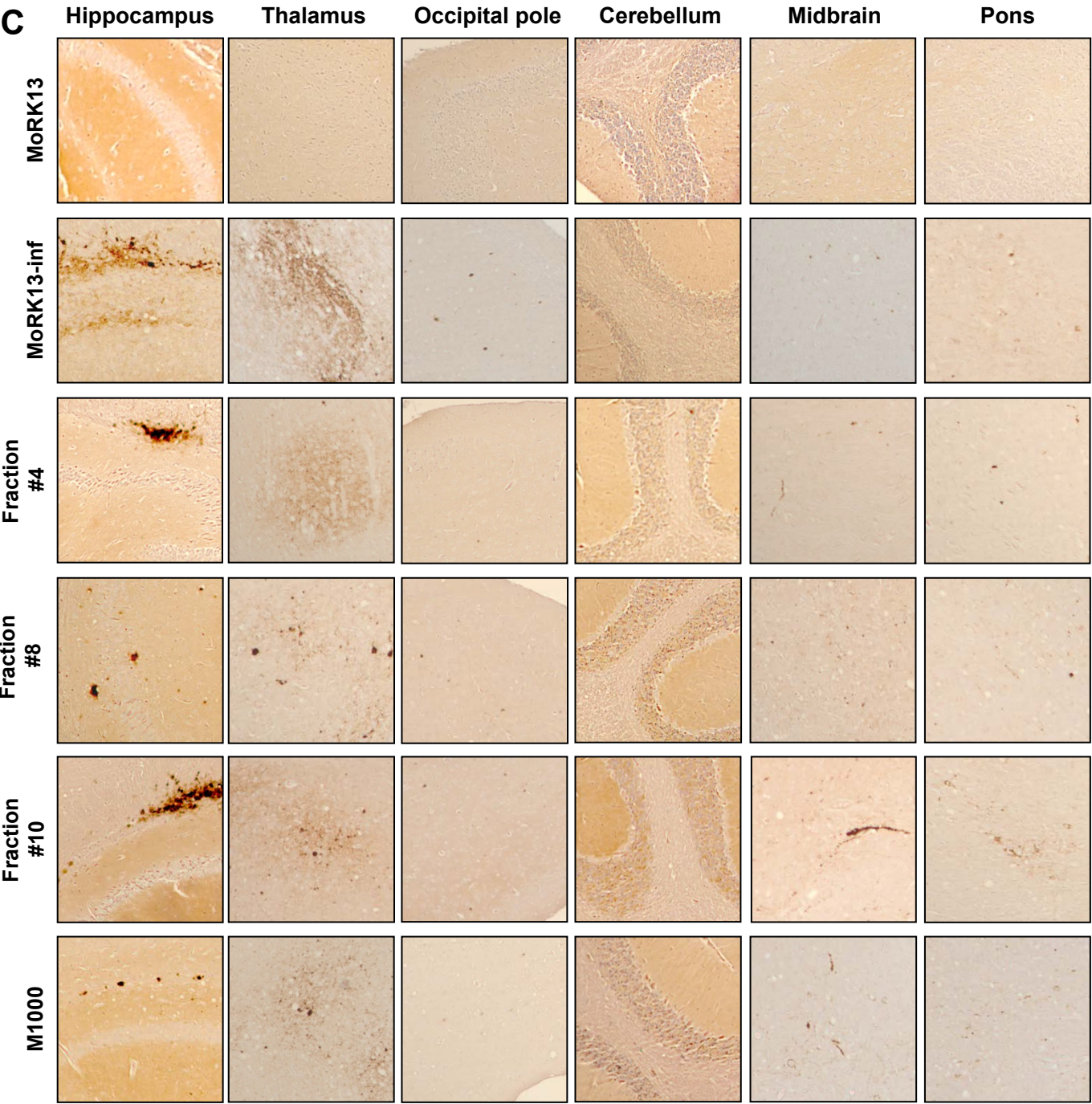

Supplement: Additional file 5 — Figure S5. Assessment of neuropathology in the brains of Tga20 mice inoculated with 0.01% (w/v) M1000 brain homogenate, MoRK13 and MoRK13-inf whole cell lysates and selected MoRK13-inf fractions. Representative photomicrographs demonstrating the degree of (A) vacuolation in hematoxylin and eosin-stained (H & E) sections, (B) astrocytic gliosis in glial fibrillary acidic protein (GFAP) stained sections, and (C) PrP plaque deposition in ICSM18 stained sections of various brain regions as indicated. Magnification = 50 × for all sections/stains except H & E stained hippocampus and occipital pole, where magnification = 100 ×. [file 1750-1326-7-18-S5.PDF]

Fig. S6

A

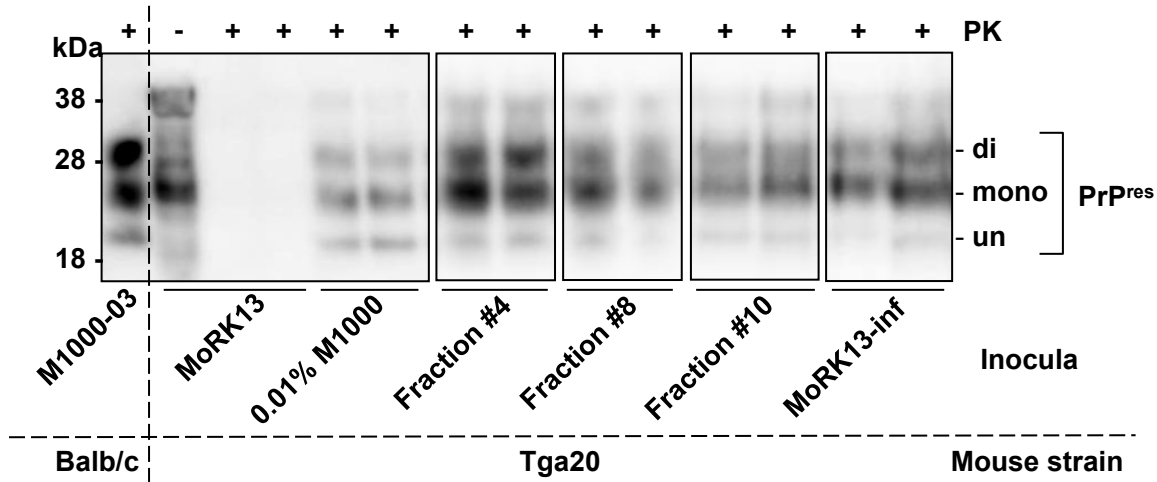

B

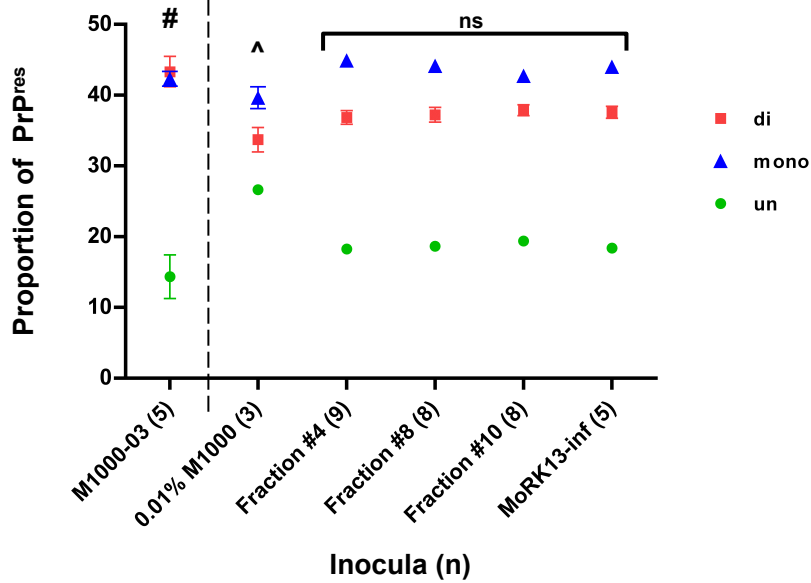

Supplement: Additional file 6 — Figure S6. PrPres profiles in Tga20 mice inoculated with 0.01% (w/v) M1000 brain homogenate, MoRK13-inf and selected MoRK13-inf subcellular fractions. Representative western blot (A) and quantification (B) of PrPres glycoform ratios in terminal mice brains after intracerebral inoculation as indicated. (A) 10 μl of PK digested (100 μg/ml final concentration PK, 1 hour at 37°C) 10% (w/v in PBS) homogenate was resolved on 12% Bis-tris NuPAGE and probed with ICSM18 primary antibody and (B) quantified using ImageJ. Di = di-glycosylated PrPres, mono = mono-glycosylated PrPres, un = unglycosylated PrPres. Statistical analysis by two-way ANOVA with Bonferroni post-tests; ns = no significant differences; #M1000 in Balb/c mice was significantly different to all other inocula; ^0.01% M1000 in Tga20 mice was significantly different to all other inocula. [file 1750-1326-7-18-S6.PDF]

Fig. S7

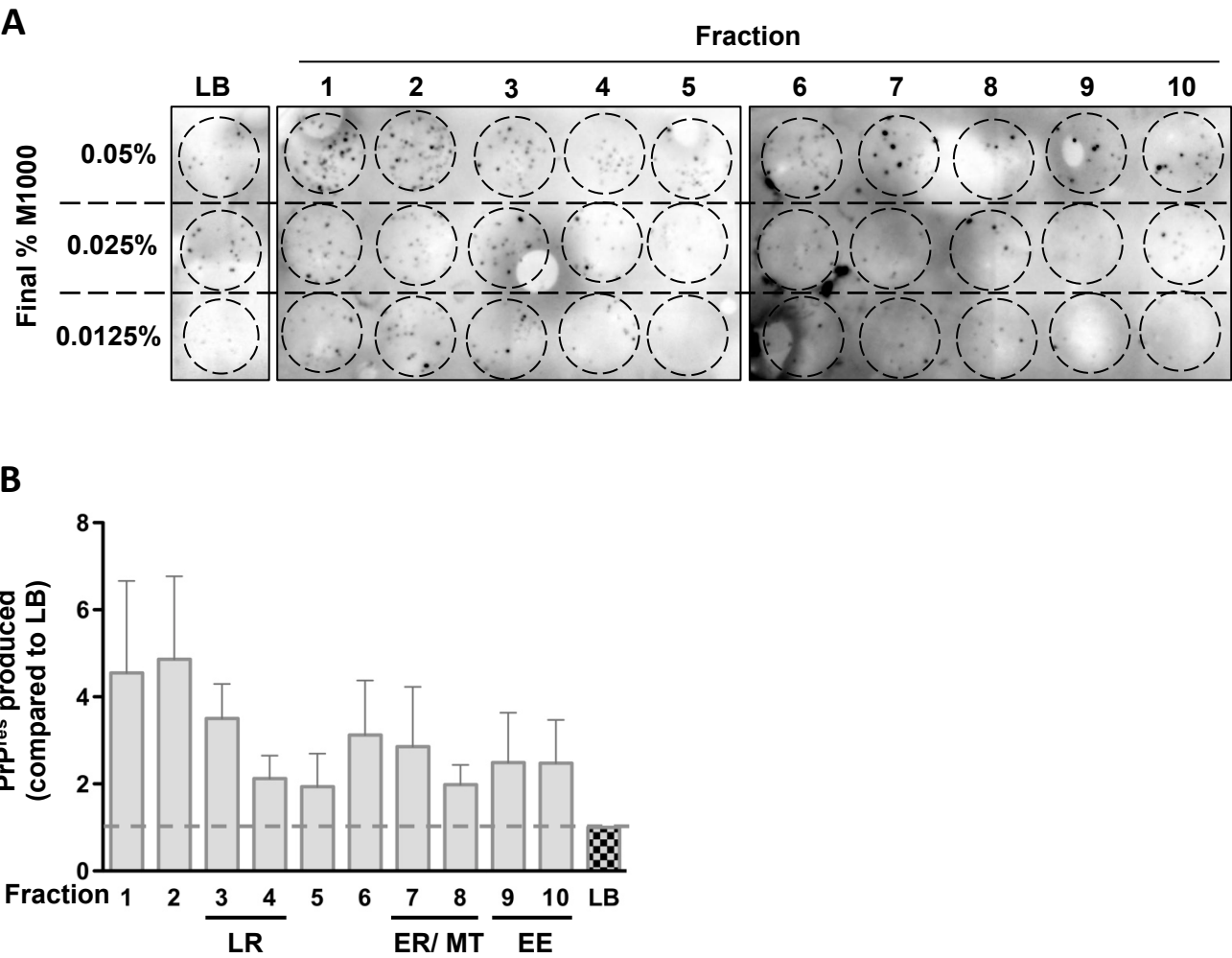

Supplement: Additional file 7 — Figure S7. Exogenous cellular co-factors from vecRK13 do not differentially increase the efficiency of M1000 infection in vitro. (A) Cell blot showing PrPres levels produced by recipient MoRK13 cells exposed to M1000 brain homogenate diluted to three different final concentrations (as indicated) with either 1:4 lysis buffer (LB):medium or 1:4 vecRK13 fraction:medium mix. (B) Quantification of PrPres produced by MoRK13 cells exposed to M1000 brain homogenate diluted in subcellular fraction relative to the equivalent % M1000 brain homogenate diluted in lysis buffer; for quantification purposes the three different % M1000 spikes into the same vecRK13 fractionated lysate were considered a triplicate of the same experiment, with the error bars representing this intra-experiment variation, and analysis of this variation (one way ANOVA) showing no significant differences. Fractions enriched in lipid raft (LR), endoplasmic reticulum (ER), mitochondrial (MT) and early endosome (EE) marker proteins are as marked. [file 1750-1326-7-18-S7.PDF]

Fig. S8

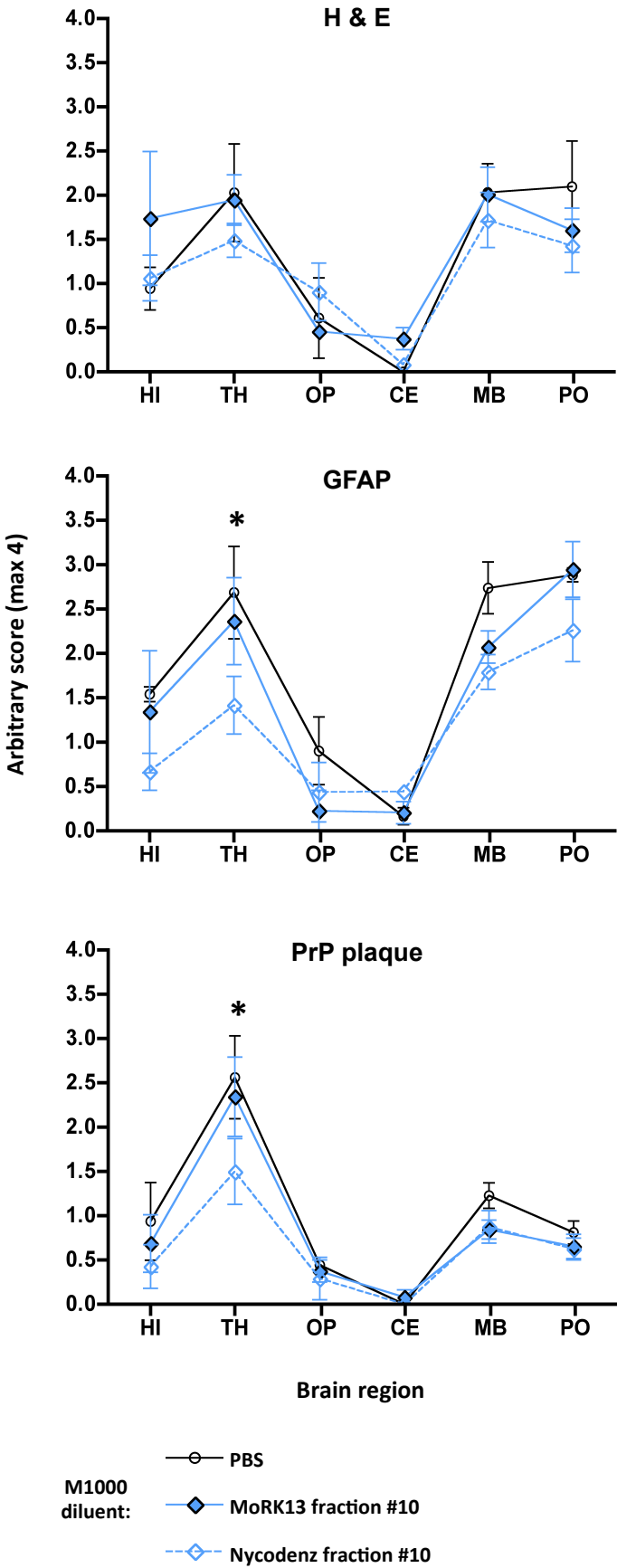

Supplement: Additional file 8 — Figure S8. Lesion profiles from Tga20 mice intracerebrally inoculated with M1000 brain homogenate diluted in PBS and high density Nyocodenz with or without MoRK13 cell lysate content. Lesion profiles generated by quantification of the degree of vacuolation (H&E), reactive astrocytosis (GFAP) and PrP plaque deposition in each brain region (HI - hippocampus, TH - thalamus, OP - occipital pole, CE - cerebellum, MB - midbrain, PO - pons). n = 3 or 4 for all brain regions except in 0.01% M1000 diluted in PBS HI PrP plaque deposition, where n = 2. Statistical analysis comparing lesion profiles was by two way ANOVA. For astrocytic gliosis and PrP plaque deposition, *p < 0.05 comparing M1000 diluted in PBS and Nycodenz fraction #10 in TH region only. No other significant differences seen. [file 1750-1326-7-18-S8.PDF]
